# Financial burden of severe childhood illness on households in Lao People’s Democratic Republic: A prospective cohort study

**DOI:** 10.1371/journal.pgph.0004783

**Published:** 2026-02-20

**Authors:** Alicia Quach, Mayfong Mayxay, Laddaphone Bounvilay, Amphaivanh Thammavong, Toukta Bounkhoun, Chom Phaiphichit, Nar Kingkeooudom, Sommanikhone Phangmanixay, Phouthalavanh Souvannasing, Elizabeth A. Ashley, Cattram Nguyen, Natalie Carvalho, Fiona M. Russell

**Affiliations:** 1 Asia-Pacific Health, Murdoch Children’s Research Institute, Melbourne, Australia; 2 Department of Paediatrics, The University of Melbourne, Melbourne, Australia; 3 Lao-Oxford-Mahosot Hospital-Wellcome Trust Research Unit (LOMWRU), Microbiology Laboratory, Mahosot Hospital, Vientiane, Lao People's Democratic Republic; 4 Unit for Health Evidence and Policy (UHEP), Institute of Research and Education Development (IRED), Lao University of Health Sciences, Ministry of Health, Vientiane, Lao People's Democratic Republic; 5 Centre for Tropical Medicine and Global Health, Nuffield Department of Clinical Medicine, University of Oxford, Oxford, United Kingdom; 6 Saw Swee Hock School of Public Health, National University of Singapore, SingaporeSingapore; 7 Department of Pediatrics, Salavan Provincial Hospital, Salavan, Lao People's Democratic Republic; 8 Department of Pediatrics, National Children’s Hospital, Vientiane, Lao People's Democratic Republic; 9 Melbourne School of Population and Global Health, The University of Melbourne, Melbourne, Australia; African Population and Health Research Center, KENYA

## Abstract

Lao People’s Democratic Republic (PDR) introduced a National Health Insurance (NHI) scheme in 2016 to all provinces except Vientiane Capital City. We describe the financial impact on households related to treatment of severe childhood illness at a hospital covered by NHI, and one without NHI. We conducted a prospective cohort study (2022–2024) in Lao PDR of children aged one month to <15 years admitted with severe illness at Salavan Provincial Hospital (SPH) with NHI, and National Children’s Hospital (NCH) without NHI, with two-month follow-up post-discharge. Illness-related direct and indirect costs were collected. We calculated household out-of-pocket (OOP) costs, impoverishment and catastrophic health expenditure (CHE, > 10% annual household expenditure) rates and analysed relative risk (RR) of CHE by socioeconomic status. 200 participants were recruited from each hospital with demographic differences observed between hospitals in urban residence (NCH 87.0%, SPH 14.5%), maternal education (primary level: NCH 95.9%, SPH 76.3%) and wealth status (wealthiest quintile: NCH 79.0%, SPH 20.5%). Median household OOP costs were higher at NCH (USD290.6 [IQR 206.9–422.9]) compared to SPH (USD92.4 [IQR 56.3–52.9]). Impoverishment at two months post-discharge was 0.5% (95%CI 0.0-3.0) at NCH and 10.2% (95%CI 6.2-15.4) at SPH. CHE rates were 34.5% (95%CI 27.9-41.1) at NCH and 26.0% (95%CI 19.9-32.1) at SPH, with higher RR in the poorest versus wealthiest households (NCH: RR 6.6, 95%CI 4.5-9.5; SPH: RR 4.9, 95%CI 1.7-13.7) and households with no formal maternal education versus secondary education (NCH: RR 2.6, 95%CI 1.2-5.5; SPH: RR 4.6, 95%CI 1.92-11.1). Direct medical costs were lower at the provincial hospital where NHI is available, but total OOP costs and CHE rates were high at both hospitals, particularly among disadvantaged households. Additional interventions are required to prevent severe illness and provide financial protection for socioeconomically disadvantaged groups to reduce health-related economic burden on households in Lao PDR.

## Introduction

Achieving universal health coverage (UHC) is a target of the Sustainable Development Goals (SDGs) and means all people can access quality essential health services without financial hardship [1]. Financial hardship can be measured as catastrophic health expenditures (CHE), when out-of-pocket (OOP) payments for healthcare exceed a certain threshold of household income; or impoverishment, when OOP payments push individuals or households below poverty lines [2–10]. Most recent figures estimated that 13.5% of the world’s population experienced CHE and 4.4% were impoverished, due to health care expenditures [11]. People living in low-and-middle-income-countries (LMICs) were almost exclusively affected, with higher rates of financial hardship occurring in the poor, those living in remote areas and marginalised groups [11].

OOP costs for inpatient care of sick children often exceed the direct medical costs of investigations, medicines and hospital bed fees [8,10,12]. A systematic review evaluating inpatient care of childhood infectious diseases in LMICs found that direct non-medical OOP costs, such as transportation and accommodation, and indirect costs (loss of productivity/income for caregivers), contributed 29% and 42% of total OOP costs, respectively, creating high financial distress on households [10].

Health insurance is one strategy used to reduce OOP health costs, but coverage in LMICs remain low with variable impact on financial hardship [13]. Lao People’s Democratic Republic (Lao PDR), a lower-middle income country in South-East Asia, where OOP expenditure has been the predominant source of health financing, launched the National Health Insurance (NHI) scheme in 2016 to regulate user fees at health facilities. With an ethnically diverse, mostly rural population of 7.6 million people, and a high national poverty rate of 18.3%, skewed towards rural households, those with less formal education, and minority ethnic groups, the NHI aims to make quality health services more accessible and affordable for vulnerable populations [14–19]. Under the NHI, essential health services are free for pregnant women, children under five years of age and the poor, with small co-payments for others [20]. The NHI covers all provinces except Vientiane Capital City, where residents continue to have access to pre-existing insurance schemes, leaving about 6% of the population not eligible for any form of health insurance [21].

There is minimal published literature on whether health insurance programs provide adequate financial protection at the consumer level in LMICs [13]. Early studies following NHI introduction in Lao PDR revealed CHE rates reaching over 30% [18,20]. To date, there have been no studies in Lao PDR evaluating levels of financial hardship or inequity gaps related to healthcare costs for sick children, in the context of the NHI. The primary aim of our study is to describe the financial impact (household OOP costs, CHE and impoverishment) on households with children with severe illnesses requiring treatment at hospitals with and without NHI. Our secondary aim is to evaluate the differences in financial burden across wealth quintile groups, geographical residence, maternal education and ethno-linguistic subpopulation groups. This will add to the limited data on financial protection and inequity gaps towards Lao PDR’s goal of achieving UHC.

## Methods

### Study design

This is a prospective cohort study of children admitted with severe illness to two different hospitals in Lao PDR, with community follow-up at two weeks and two months following discharge. Data on health-related OOP costs were collected from a household perspective. The study was reported using the Strengthening the Reporting of Observational Studies in Epidemiology (STROBE) Statement [22]. *S1 Checklist* *provides details for this study using the STROBE Checklist for Cohort Studies.*

### Setting

Lao PDR consists of 17 provinces and one prefecture, Vientiane Capital City. Nationally, there are eight central, tertiary level hospitals, 17 provincial, secondary level hospitals and 135 district hospitals [19].

Participants were recruited from two hospitals: National Children’s Hospital (NCH), a central, tertiary level hospital with 70 paediatric beds in Vientiane Capital City not covered by NHI; and Salavan Provincial Hospital (SPH), a provincial, secondary level hospital with 49 paediatric beds in Salavan Province in Southern Lao PDR, fully covered by NHI. In 2023, NCH had 11,418 paediatric admissions and SPH had 3550 paediatric admissions.

### Participants

We enrolled children aged between one month of age and <15 years old, presenting to either NCH or SPH with acute symptoms starting within 14 days prior to hospital admission, and diagnosed with a severe illness. A severe illness was defined as showing emergency signs as per WHO Pocketbook criteria and/or requiring hospital admission for further treatment [23]. Emergency signs included: obstructed or absent breathing, severe respiratory distress, central cyanosis, shock, coma, convulsions or severe dehydration [23].

We excluded neonates and those admitted to the neonatal intensive care unit (NICU), as they are a specific population that would likely skew results. We also excluded children admitted for planned procedures or treatment of chronic medical conditions to focus on OOP costs for acute severe medical events with unanticipated household costs. Parents/guardians of enrolled children were required to speak the Lao language and be contactable by phone.

### Study procedures

For each participant, four study visits were conducted – at enrolment (hospital admission), at hospital discharge, and then at two weeks and two months following hospital discharge. Study visits were conducted between 28 October 2022 and 10 January 2024. Informed consent procedures were conducted at enrolment. As all participants were under 18 years, written consent was obtained from a parent/legal guardian of the participant. Signed assent was also sought from participants age 12 – 15 years, assessed as physically well enough and having the mature capacity to understand the study. All study visits were conducted in-person with the parent/caregiver and child, except at two weeks post-discharge, where a phone interview was conducted. Those who could not attend in-person for the study visit at two months post-discharge, were contacted for a phone interview.

Study staff recorded data on paper-based questionnaires with the parent/caregiver and directly with the child, where appropriate. Information collected included household demographics and socioeconomic status (age of child, ethnicity, parental education levels and occupation, estimated income, housing materials and assets), household expenditures (general expenditures as well as OOP costs related to the child’s current illness), financial coping strategies (health insurance, savings, borrowing money, selling assets) and the child’s physical health outcome post-discharge (recovery status, death). Information on the child’s inpatient management (admitted ward, duration of stay, diagnosis and discharge destination) were collected from hospital medical records.

Household consumption data were collected as a recall of expenses over a two-week period, prior to enrolment, and prior to the two-week and two-month post-discharge study visits. A two-week period was used to minimise recall bias and align with the most recent Lao Expenditure Consumption Survey 6 (LECS6) methodology [17]. Expenses collected included food, housing, utility bills, fuel, transportation, education, clothing, personal care, recreation, and medical expenditures for other household members. Data were used to estimate household expenditures over two months and over one year.

Data collected at each study visit on household OOP costs associated with the child’s current illness included: direct medical costs (medical consultation fees, diagnostics, medicines, hospital bed fees, hospital transfer costs and insurance co-pay if applicable); direct non-medical costs (travel costs for the patient and accompanying carers/household members, daily living expenses whilst in hospital such as food, accommodation, childcare costs not usually paid for other children); and indirect costs (estimated loss of income of the primary income earner and primary caregiver due to child’s illness). This was based on the caregiver reported estimated daily income for the primary income earner and primary caregiver and calculated as the sum of (reported daily income x days of productivity lost).

### Outcome definitions

#### Out-of-pocket costs.

The primary outcome of interest was the total household OOP costs related to the severe illness. This was calculated by adding direct (medical and non-medical) costs less any reimbursements such as health insurance, with indirect costs, incurred from the onset of symptoms to two months post-discharge from hospital. OOP costs were calculated using the local currency, Lao Kip (LAK) and then converted to United States Dollars (USD) using the official World Bank exchange rate for 2023 of LAK 17689 = USD 1 [24].

#### Catastrophic health expenditures.

A household was deemed to have CHE if OOP costs exceeded a certain percentage of household expenditures or capacity-to-pay, where capacity-to-pay was defined as the total household expenditures minus necessary subsistence expenditures (food, housing and utilities). We used four different thresholds to define CHE: 10% of two-monthly HE and 40% of two-monthly capacity-to-pay were used to assess short-term financial impacts; 10% of annual household expenditures and 40% of annual capacity-to-pay to assess longer-term financial impacts of OOP costs. We chose 10% of annual household expenditures to align with recommended indicators for SDG monitoring [1,2]. 40% of annual capacity-to-pay was also used, as an alternative for CHE calculation deemed more sensitive at identifying poorer populations [2,4,5,25–27]. As our study only recruited children with acute illnesses, we included short-term financial impacts over two months, to reflect the acute nature of the illnesses [2,10,26].

#### Impoverishment.

Two definitions for impoverishment were used: the International Poverty Line set at $2.15/person/day at 2017 PPP and the Laos National Poverty Line set at LAK 280,910/person/month at 2019 local prices (USD 22.00/person/month), both adjusted for inflation [11,17]. The international poverty line was used to align with SDG indicators and enable cross-country comparisons, and the national poverty line to be more representative of poverty within Lao PDR [9,11]. Household expenditure was used as a proxy for household income in Lao PDR where households are mainly supported by farming and subsistence living and not formal salaries. A household was deemed impoverished if the household expenditure excluding the total direct (medical and non-medical) OOP costs, divided by the household headcount (converted to daily and monthly expenditures per person), was below the poverty line.

### Study size

The sample size was based on the primary objective of estimating average household OOP costs across both hospitals related to treatment of children with a severe illness in Lao PDR. We extended calculations to also allow comparison of OOP costs across wealth quintiles. A sample size of 60 participants per quintile was needed to obtain 90% power and a significance level of 0.05 to detect a mean difference of LAK 82,000 (USD 8.5) across successive wealth quintiles (based on population data from previous analyses of the Lao Expenditure Consumption Survey) [28]. Accounting for 20% loss to follow up, and presumed equal distribution across the wealth quintiles, the total sample size was 375 across both hospitals.

### Statistical methods

Participant demographics, primary clinical diagnoses, inpatient management and health outcomes were summarised by hospital. Categorical variables were summarised as frequencies and percentages of participants, and continuous variables with medians (IQR). Each household was assigned to a wealth quintile by first creating a wealth index score using Principal Components Analysis using 77 household demographics and consumption variables, where factor weights were equivalent to those used in the Laos Social Indicator Survey II (LSIS II) [29]. Participants were then classified into wealth quintiles using thresholds for the national wealth quintiles [29,30].

For our primary aim, OOP costs, CHE and impoverishment rates were summarised by hospital. Medians (IQR) and means (SD) were calculated for OOP costs. Mean CHE and impoverishment percentage rates were calculated with 95% confidence intervals (CIs). We calculated impoverishment rates at baseline, two weeks and two months post-hospital discharge to determine if there were ongoing financial impacts from the severe illness.

For our secondary aim, OOP costs, CHE and impoverishment rates were disaggregated by wealth quintiles, maternal education categories, geographical residence and the four formal ethno-linguistic groups. Differences in median OOP costs across subgroups were estimated using quantile regression. Equity analyses included comparing risk ratios (RR) of CHE and impoverishment rates across the subpopulation groups, by hospital. Due to non-convergence, RR were estimated using Poisson regression with robust standard errors.

Data were entered and managed on REDCap electronic data capture software platform with manual second person data entry checks and inbuilt range checks for data cleaning prior to analysis. All statistical analyses were performed using statistical software package STATA (v18.0) [31].

### Ethical clearance

The study was conducted according to the protocol approved by The Royal Children’s Hospital Human Research Ethics Committee (HREC 81864/RCHM-2022), the University of Oxford Tropical Research Ethics Committee (552–22), and the Lao PDR Ministry of Health, National Ethics Committee for Health Research (2022.52). Additional information regarding the ethical, cultural and scientific considerations specific to inclusivity in global research is included in the Supporting Information (S2 Checklist).

## Results

**Fig 1:**
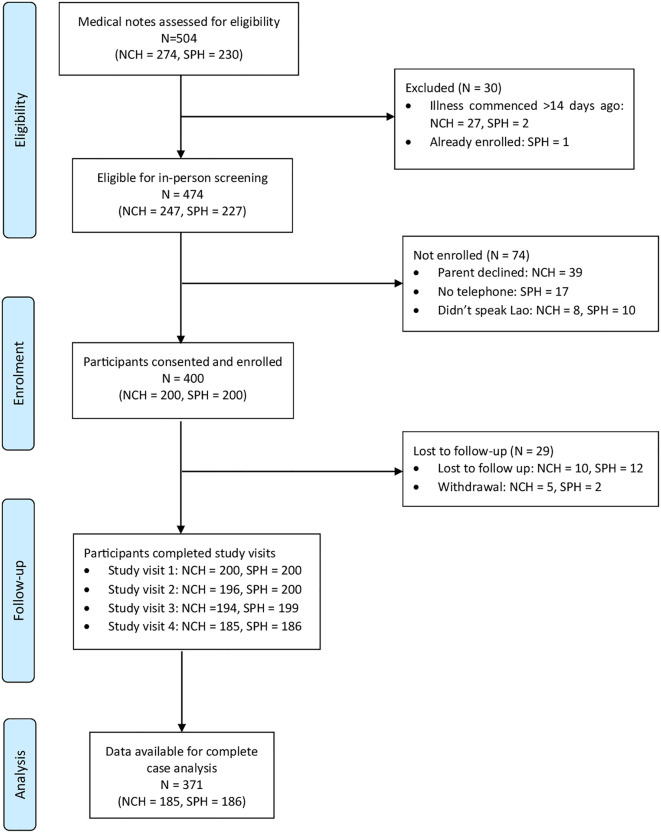
Study flowchart of participant recruitment and follow-up.

Of the 474 children screened, a total of 400 participants were enrolled into the study, 200 from each hospital. Reasons for not being enrolled included parent decline, not having a telephone and not speaking the Lao language (Fig 1). Preliminary analysis after reaching the calculated sample size of 375 revealed participants were skewed towards the wealthier quintiles (Q4 and Q5). Recruitment therefore continued to try to reach the target of 60 participants for each wealth quintile, but was ceased before attaining this, deemed unlikely to be reached within a reasonable time period. 371 (92.8%) participants completed all four study visits with data available for complete case analysis.

Table 1 summarises the demographic characteristics of participants and household members, by hospital. The median age of participants was one year (IQR 0.3 – 3.7 years) with most (82%) below five years of age. Characteristics differed between the two hospitals: almost all households (97.5%) from NCH were from the two wealthiest quintiles (Q4 and Q5), whereas households from SPH were more evenly distributed across wealth quintiles. Most households from NCH lived in urban areas (87%) whereas most from SPH lived in rural areas (85.5%). Mothers from NCH had higher education levels, with 85.1% having completed secondary level or higher, compared to SPH where 23.7% of mothers having had no formal education. All households from SPH were covered by the NHI. At NCH, 36.5% of households had some form of health insurance, mostly from the formal sector covering civil servants and private formal employees.

**Table 1:** Demographic characteristics of participants and household members.

	National Children’s Hospital N (%)	Salavan Provincial Hospital N (%)
**Number of participants**	N = 200	N = 200
**Age of participant, in years**		
Median (IQR)	0.9 (0.3, 2.4)	1.4 (0.5, 4.9)
**Age group**		
1–11 months	110 (55%)	86 (43%)
12–59 months	66 (33%)	65 (32.5%)
60 months to <15 years	24 (12%)	49 (24.5%)
**Sex (participant)**		
Male	104 (52.0%)	103 (51.5%)
Female	96 (48.0%)	97 (48.5%)
**Geographical residence**		
Urban	174 (87.0%)	29 (14.5%)
Rural with road access	26 (13.0%)	171 (85.5%)
**Ethno-linguistic group**		
Lao-Tai	173 (86.5%)	168 (84.0%)
Mon-Khmer	6 (3.0%)	29 (14.5%)
Hmong-Mien	19 (9.5%)	0 (0.0%)
Chinese-Tibetan	1 (0.5%)	0 (0.0%)
Other	1 (0.5%)	3 (1.5%)
**Household number**		
Median (range)	5 (3-14)	5 (3-10)
**Relationship of respondent/guardian to participant**		
Mother/stepmother	160 (80.0%)	179 (89.5%)
Father/stepfather	33(16.5%)	13 (6.5%)
Other	7 (3.5%)	8 (4.0%)
**Mother’s education level**		
None/early childhood	8 (4.1%)	45 (23.7%)
Primary	21 (10.8%)	62 (32.6%)
Secondary or higher	165 (85.1%)	83 (43.7%)
**Occupation of primary income earner**		
Farmer/ agricultural	9 (4.5%)	126 (63.0%)
Manual laborer	136 (68.0%)	59 (29.5%)
Service and sales	47 (23.5%)	12 (6.0%)
Professional	4 (2.0%)	0 (0%)
House duties	3 (1.5%)	2 (1.0%)
Unemployed	1 (0.50%)	1 (0.5%)
**Wealth quintile (household)**		
Q1 (poorest)	0 (0.0%)	21 (10.5%)
Q2	3 (1.5%)	38 (19.0%)
Q3	2 (1.0%)	50 (25.0%)
Q4	37 (18.5%)	50 (25.0%)
Q5 (wealthiest)	158 (79.0%)	41 (20.5%)
**Covered by health insurance**		
Yes	73 (36.5%)	200 (100%)
**Type of health insurance (if covered),** may select more than one if applicable		
National Health Insurance (NHI)	0 (0%)	198 (99%)
Civil servant	29 (39.7%)	0 (0%)
Social security	40 (54.8%)	0 (0%)
Community based health	0 (0%)	0 (0%)
Health equity fund	0 (0%)	0 (0%)
Free maternal child health	1 (1.4%)	0 (0%)
Private health	5 (6.8%)	2 (1%)
Other	1 (1.4%)	0 (0%)

### Inpatient management and health outcomes

Table 2 summarises the inpatient management and health outcomes of the participants. The length of hospital stay was longer at NCH: median 6 days (IQR 4–7 days) compared to SPH: median 4 days (IQR 3–5 days). The most common primary clinical diagnosis at both hospitals was lower respiratory tract infection or pneumonia (NCH 62.3%, SPH 35.5%). 10.2% of participants from NCH went home before completing treatment, compared to 3.0% from SPH. Across both hospitals, five participants died whilst in hospital, with a further eight participants dying after leaving hospital.

**Table 2:** Clinical diagnoses, inpatient management and health outcomes.

	National Children’s Hospital N (%) N = 199	Salavan Provincial Hospital N (%) N = 200
**Ward admitted to**		
Pediatric General Medicine	136 (68.4%)	154 (77.0%)
Pediatric Intensive Care Unit (ICU)	63 (31.7%)	46 (23.0%)
**Length of hospital admission, days**		
Median (IQR)	6 (4-7)	4 (3-5)
**Primary clinical diagnosis**		
Lower respiratory tract infection (pneumonia)	124 (62.3%)	71 (35.5%)
Upper respiratory tract infection	0 (0.0%)	43 (21.5%)
Acute gastroenteritis and dysentery	18 (9.0%)	16 (8.0%)
Dengue	13 (6.5%)	23 (11.5%)
Other infection	10 (5.1%)	15 (7.5%)
Febrile seizure	17 (8.5%)	6 (3.0%)
Beriberi	1 (0.5%)	7 (3.5%)
Other	16 (8.0%)	19 (9.5%)
**Discharge destination**		
Home, treatment completed	172 (86.4%)	189 (94.5%)
Home, treatment not completed	20 (10.2%)	6 (3.0%)
Transfer to another health facility	2 (1.0%)	4 (2.0%)
Deceased	4 (2.0%)	1 (0.5%)
Other	1 (0.5%)	0 (0.0%)
Health outcomes post-discharge*		
Fully recovered	146 (76.8%)	114 (60.3%)
Partially recovered, improved since discharge	33 (17.4%)	69 (36.5%)
Not recovered, same or sicker since discharge	2 (1.1%)	2 (1.1%)
Deceased (inclusive of deaths in hospital)	9 (4.7%)	4 (2.1%)

* Complete case analysis: n = 379.

### Out-of-pocket costs

The median household OOP costs from the onset of illness to two months after hospital discharge were summarised by hospital (Table 3). Both mean and median OOP costs were calculated, however due to the skewed distribution of OOP costs and a small number of extreme outliers with very high OOP costs biasing the mean, we used the median OOP costs to compare differences between groups. *A summary of mean household OOP costs and detailed itemised costs are included in S1 Table and S2 Table*, *respectively.* The median total household OOP costs including all direct and indirect costs was over three times higher at NCH: USD290.6 (IQR 206.9 – 422.9) than it was at SPH: USD92.4 (IQR 56.2 – 152.9). Median direct medical OOP costs at NCH was USD128.9 (IQR 70.1 – 218.2) compared with SPH which was USD3.4 (IQR 0.0 – 14.1). There were several outliers, with nine households (six from NCH, three from SPH) incurring more than USD1000 in total OOP costs.

**Table 3:** Out-of-pocket costs (in USD) associated with severe illness, by hospital.

	National Children’s Hospital N = 185*	Salavan Provincial Hospital N = 186*
**Direct medical OOP costs, median (IQR)**	128.9 (70.1–218.2)	3.4 (0.0–14.1)
**Direct non-medical OOP costs, median (IQR)**	65.0 (45.2–82.54)	48.9 (37.9–80.3)
**Indirect OOP costs, median (IQR)**	84.8 (45.2–132.8)	25.4 (10.2–67.8)
**Total OOP** **(direct medical + direct non-medical costs, median (IQR))**	195.6 (128.9–285.5)	58.0 (40.1–96.1)
**Total OOP** **(direct + indirect costs, median (IQR))**	290.6 (206.9–422.9)	92.4 (56.2–152.9)

OOP = out-of-pocket; USD = United States Dollar.

* Complete case analysis (i.e., Data were available for all study visits).

Subgroup analyses (Table 4) revealed that when comparing total OOP costs by wealth subgroups within each hospital, at SPH the wealthiest quintile (Q5) had greater OOP costs than the poorest quintile (Q1): (difference in median OOP costs: USD 69.5, 95% CI 17.3 – 121.7, p = 0.009). Other subgroup analyses showed OOP costs were similar by geographical residence, maternal education and ethno-linguistic groups by hospital.

**Table 4:** Comparison of total out-of-pocket costs^^^ (in USD) associated with severe illness, by demographics.

	National Children’s Hospital (N = 185*)				Salavan Provincial Hospital (N = 186*)			
	N (%)	OOP costs^^^, median (IQR)	∆ medians (95% C.I.)#	P-value	N (%)	OOP costs^^^, median (IQR)	∆ medians (95% C.I.)#	P-value
**Wealth Quintile**								
**Q1 (Poorest)**	0	N/A	−	−	17 (9.2%)	63.9 (45.8, 112.0)	−69.5 (−121.7, −17.3)	0.009
**Q2**	0	N/A	−	−	34 (18.3%)	94.4 (48.6, 161.12)	−37.3 (−79.5, 4.9)	0.082
**Q3**	1 (0.5%)	110.2 (110.2, 110.2)	−179.2 (−546.7, 188.3)	0.337	46 (25.3%)	90.4 (56.8, 152.9)	−40.1 (−79.2, −1.0)	0.044
**Q4**	35 (18.9%)	294.0 (221.9, 466.1)	4.5 (−64.3, 73.3)	0.897	50 (26.9%)	83.1 (56.2, 134.3)	−47.5 (−85.9, −9.1)	0.016
**Q5 (Wealthiest)**	149 (80.5%)	289.4 (203.5, 420.9)	Ref	Ref	39 (21.0%)	133.4 (72.9, 235.7)	Ref	Ref
**Geographical residence**								
**Urban**	165 (89.2%)	285.5 (198.7, 422.9)	Ref	Ref	27 (14.5%)	87.1 (54.27, 216.5)	Ref	Ref
**Rural**	20 (10.8%)	334.1 (247.1, 401.4)	48.6 (−42.3, 139.5)	0.293	159 (85.5%)	93.3 (56.2, 150.4)	6.6 (−33.9, 46.3)	0.760
Maternal education*								
**None/** **early childhood**	7 (3.8%)	342.9 (212.0, 466.1)	54.3 (−89.7, 198.2)	0.458	37 (19.9%)	85.9 (53.7, 129.2)	−7.3 (−47.5, 32.8)	0.718
**Primary**	20 (10.8%)	298.6 (235.3, 379.8)	14.9 (−73.7, 103.4)	0.741	58 (31.2%)	102.3 (48.6, 183.7)	13.8 (−20.9, 48.6)	0.433
**Secondary or higher**	154 (83.3%)	287.0 (203.6, 422.9)	Ref	Ref	82 (44.1%)	92.7 (61.9, 188.2)	Ref	Ref
**Ethno-linguistic group**								
**Lao-Tai**	162 (87.6%)	292.6 (208.0, 444.2)	Ref	Ref	158 (85.0%)	91.3 (55.4, 148.7)	Ref	Ref
**Mon-Khmer**	5 (2.7%)	212.0 (147.8, 221.9)	−81.7 (−254.9, 91.5)	0.353	25 (13.4%)	96.1 (56.8, 183.7)	4.0 (−36.3, 44.2)	0.846
**Hmong-Mien**	16 (8.6%)	292.3 (187.0, 436.8)	0.3 (−99.7, 100.3)	0.996	0	N/A	N/A	N/A
**Chinese-Tibetan**	1 (0.5%)	248.2 (248.2, 248.2)	−45.5 (−428.2, 337.2)	0.815	0	N/A	N/A	N/A
**Other**	1 (0.5%)	313.7 (313.7, 313.7)	20.1 (−362.6, 402.8)	0.918	3 (1.6%)	97.8 (65.6, 199.0)	5.6 (−103.3, 114.6)	0.919

^ OOP costs = total (direct + indirect) costs associated with severe illness over whole study period.

* Complete case analysis (data were available for all study visits). NB for maternal education group – 13 missing values.

# Quantile regression analysis to compare differences in median OOP costs across subgroups.

### Catastrophic health expenditures and impoverishment

Table 5 summarises the combined direct and indirect costs calculated as a percentage of household expenditures. At both hospitals, the total OOP costs consumed more than half of the two-monthly household expenditure (NCH: 55.9%, 95% CI 47.3 – 64.4; SPH: 53.3%, 95% CI 37.2 – 69.5). The total OOP costs as a percentage of annual household expenditures was 9.3% (95% CI 7.9 – 10.7) at NCH and 8.9% (95% CI 6.2 – 11.6) at SPH. There were differences between the two hospitals when only direct medical costs were considered, but these differences were less when direct non-medical costs and indirect costs were included in the OOP calculation. Similar results were found when OOP costs were calculated as a percentage of capacity-to-pay.

**Table 5:** Total out-of-pocket costs as a percentage of household expenditure, by hospital.

	National Children’s Hospital N = 183	Salavan Provincial Hospital N = 186
**OOP costs as a percentage of two monthly HE – % (95% C.I.)**		
**Direct medical costs**	30.1% (23.8–36.4)	10.6% (0.0–22.7)
**Direct (medical + non-medical) costs**	40.7% (33.5–47.9)	38.0% (22.6–53.3)
**Direct + indirect costs**	55.9% (47.3–64.4)	53.3% (37.2–69.5)
**OOP costs as a percentage of two monthly CTP – % (95% C.I.)**		
**Direct medical costs**	35.3% (28.3–42.4)	12.2% (0.0–26.4)
**Direct (medical + non-medical) costs**	47.8% (39.8–55.9)	43.2% (25.1–61.3)
**Direct + indirect costs**	65.6% (56.0–75.2)	60.4% (41.4–79.4)
**OOP costs as a percentage of annual HE – % (95% C.I.)**		
**Direct medical costs**	5.0% (4.0–6.1)	1.8% (0.0–3.8)
**Direct (medical + non-medical) costs**	6.8% (5.6–8.0)	6.3% (3.8–8.9)
**Direct + indirect costs**	9.3% (7.9–10.7)	8.9% (6.2–11.6)
**OOP costs as a percentage of annual CTP – % (95% C.I.)**		
**Direct medical costs**	5.9% (4.7–7.1)	2.0% (0.0–4.4)
**Direct (medical + non-medical) costs**	8.0% (6.6–9.3)	7.2% (4.2–10.2)
**Direct + indirect costs**	10.9% (9.3–12.5)	10.1% (6.9–13.2)

OOP = out-of-pocket; HE = household expenditures; CTP = capacity to pay.

**Fig 2:**
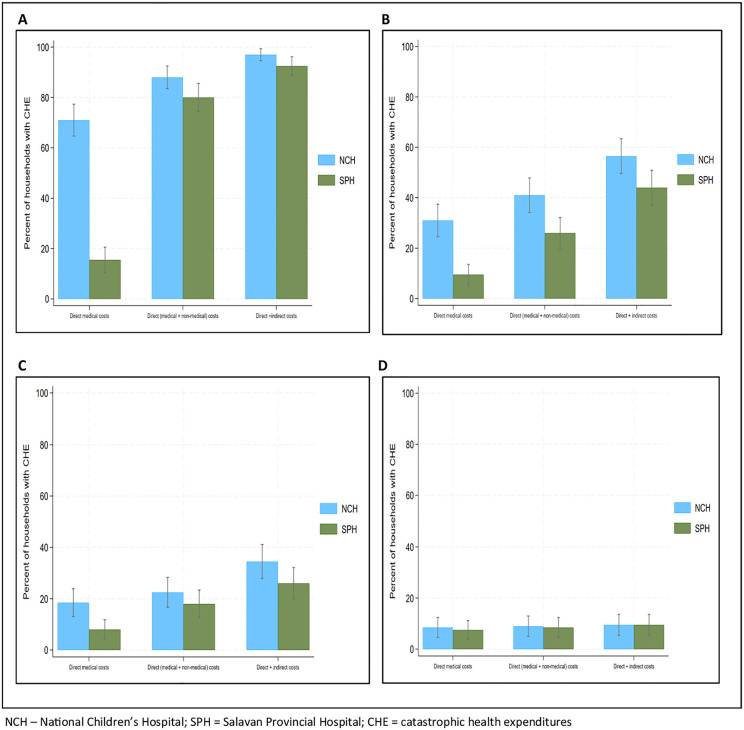
Catastrophic health expenditure rates due to severe illness. **A.** Threshold: 10% of two-monthly household expenditures. **B.** Threshold: 40% of two-monthly capacity to pay. **C.** Threshold: 10% of annual household expenditures. **D.** Threshold: 40% of annual capacity to pay. NCH – National Children’s Hospital; SPH = Salavan Provincial Hospital; CHE = catastrophic health expenditures.

When comparing CHE rates between the two hospitals, NCH had higher rates of estimated short-term financial hardship (over two months), for all OOP cost categories (Fig 2A and 2B). Estimated longer-term financial impact (over one year) only saw a large difference between the two hospitals when using direct medical costs at a threshold of 10% of annual household expenditures (NCH: 18.5%, 95% CI 13.1 – 23.9; SPH: 8.0%, 95% CI 4.2 – 11.7) with minimal differences at threshold of 40% annual capacity-to-pay (Fig 2C and 2D). *S3 Table* *provides more detailed results of CHE rates by hospital.*

**Fig 3:**
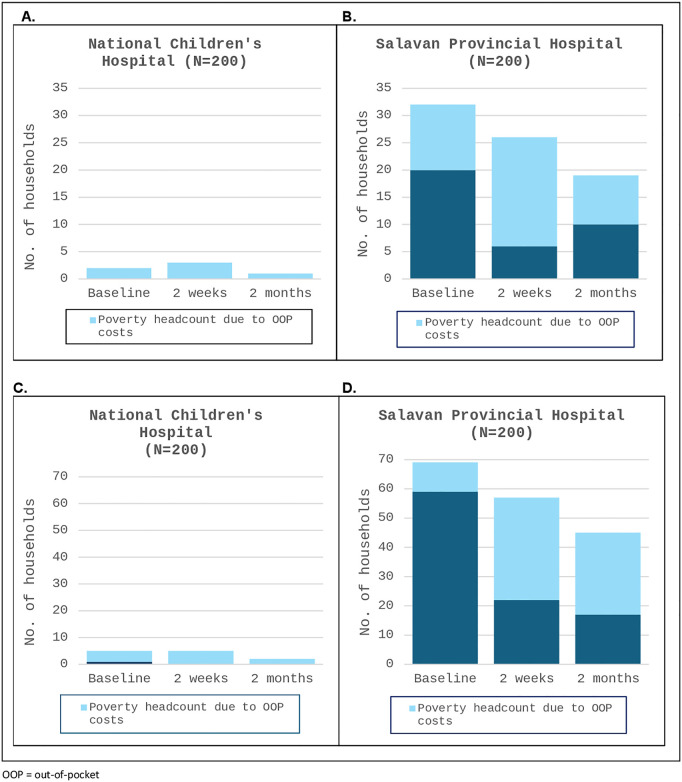
Poverty headcount associated with direct (medical & non-medical) out-of-pocket costs. **A.** National Children’s Hospital – threshold: International poverty line ($USD 2.15/person/day at 2017 PPP). **B.** Salavan Provincial Hospital – threshold: International poverty line ($USD 2.15/person/day at 2017 PPP). **C.** National Children’s Hospital – threshold: Laos national poverty line ($USD 22.00/person/month). **D.** Salavan Provincial Hospital – threshold: Laos national poverty line ($USD 22.00/person/month). OOP = out-of-pocket.

Fig 3 shows the poverty headcount associated with the OOP costs by hospital, based on both international and national poverty lines. From enrolment to two months post-discharge, less than five NCH households were impoverished due to OOP costs at both poverty thresholds (Fig 3A and 3C). Based on the international poverty line, 32 (16%) households from SPH were already in poverty at enrolment, with 12 (6%) due to OOP costs before hospital admission. At two months post-discharge, 19 (10.2%) SPH households remained in poverty (Fig 3B). Using the national poverty line, there were higher impoverishment rates from SPH, 69 (34.5%) households at enrolment and 45 (24.1%) at two months post-discharge, with 28 (15%) households impoverished due to OOP costs (Fig 3D). *S4 Table* *provides more detailed results of impoverishment rates by hospital.*

Table 6 and Table 7 summarise the CHE rates by demographic groups at each hospital, with risk ratios calculated across the subgroups. At NCH, higher rates of CHE based on threshold of 10% of annual household expenditures were seen in households from the second poorest quintile (Q2) compared to the wealthiest quintile (Q5) (RR 6.6, 95% CI 4.5 – 9.5), those living in rural versus urban areas (RR 2.4, 95% CI 1.4 – 4.1) and with no formal maternal education compared to secondary education or higher (RR 2.6, 95% CI 1.2 – 5.5). At SPH, higher rates of CHE were similarly seen from the poorest quintile (RR 4.9, 95% CI 1.7 – 13.7), and with no formal maternal education versus secondary education (RR 4.6, 95% CI 1.9 – 11.1), with some evidence of higher CHE rates in rural compared to urban areas (RR 1.9, 95% CI 0.6 – 5.7). Ethnolinguistic group analysis found higher CHE rates amongst the Mon-Khmer group (RR 3.0, 95% CI 1.7 – 5.4) compared to the Lao-Tai group. Refer to Table 6 and Table 7 for further results of CHE rates across subgroups using other thresholds.

**Table 6:** Catastrophic health expenditure rates based on direct (medical + non-medical) costs, by demographics at National Children’s Hospital.

CHE Threshold	10% of 2 monthly HE		40% of 2 monthly CTP		10% of annual HE		40% of annual CTP	
	N (%)	RR (95% C.I.)^#^	N (%)	RR (95% C.I.)^#^	N (%)	RR (95% C.I.)^#^	N (%)	RR (95% C.I.)^#^
**Wealth Quintile**								
**Q1 - Poorest** (n = 0)	N/A	N/A	N/A	N/A	N/A	N/A	N/A	N/A
**Q2** (n = 3)	3 (100%)	1.2 (1.1–1.3)	3 (100%)	3.0 (2.4–3.8)	3 (100%)	6.6 (4.5–9.5)	3 (100%)	13.2 (7.6–22.7)
**Q3** (n = 2)	2 (100%)	1.2 (1.1–1.3)	1 (50.0%)	1.5 (0.4–6.2)	1 (50.0%)	3.3 (0.8–13.9)	1 (50.0%)	6.6 (1.5–29.3)
**Q4** (n = 37)	37 (100%)	1.2 (1.1–1.3)	26 (70.3%)	2.1 (1.6 -2.9)	17 (46.0%)	3.0 (1.8–5.0)	2 (5.4%)	0.7 (0.2–3.1)
Q5*- Wealthiest (n = 158)	134 (84.1%)	Ref	52 (32.9%)	Ref	24 (15.2%)	Ref	12 (7.6%)	Ref
**Geographical residence**								
Urban* (n = 174)	151 (86.8%)	Ref	65 (37.4%)	Ref	33 (19.0%)	Ref	12 (6.9%)	Ref
**Rural** (n = 26)	25 (96.2%)	1.1 (1.0–1.2)	17 (65.4%)	1.7 (1.2–2.5)	12 (46.2%)	2.4 (1.4–4.1)	6 (23.1%)	3.4 (1.4–8.2)
**Maternal education**								
**None/ early** **childhood** (n = 8)	7 (87.5%)	1.0 (0.8–1.3)	4 (50.0%)	1.4 (0.7–2.8)	4 (50.0%)	2.6 (1.2–5.5)	9 (20.0%)	1.5 (0.2–9.9)
**Primary** (n = 21)	20 (95.2%)	1.1 (1.0–1.2)	14 (66.7%)	1.8 (1.3–2.6)	6 (28.6%)	1.5 (0.7–3.1)	5 (8.1%)	0.6 (0.1–4.1)
**Secondary or higher** (n = 165)	143 (86.7%)	Ref	60 (36.4%)	Ref	32 (19.4%)	Ref	2 (2.4%)	Ref
**Ethno-linguistic group**								
Lao-Tai* (n = 173)	150 (86.7%)	Ref	70 (40.5%)	Ref	37 (21.4%)	Ref	14 (8.1%)	Ref
**Mon-Khmer** (n = 6)	6 (100%)	1.2 (1.1–1.2)	2 (33.3%)	0.8 (0.3–2.6)	1 (16.7%)	0.8 (0.1–4.8)	1 (16.7%)	2.1 (0.3–13.3)
**Hmong-Mien** (n = 19)	19 (100%)	1.2 (1.1–1.2)	9 (47.4%)	1.2 (0.7–1.9)	7 (36.8%)	1.7 (0.9–3.3)	3 (15.8%)	1.9 (0.6–6.2)
**Chinese-Tibetan** (n = 1)	0	–	0	–	0	–	0	–
**Other** (n = 1)	1 (100%)	1.2 (1.1–1.2)	1 (100%)	2.5 (2.1–3.0)	0	–	0	–

CHE = catastrophic health expenditure; HE = household expenditure; CTP = capacity to pay.

* Reference group.

# Due to non-convergence, RR estimated using Poisson regression with robust standard errors.

**Table 7:** Catastrophic health expenditure rates based on direct (medical + non-medical) costs, by demographics at Salavan Provincial Hospital.

CHE Threshold	10% of 2 monthly HE		40% of 2 monthly CTP		10% of annual HE		40% of annual CTP	
	N (%)	RR (95% C.I.)^#^	N (%)	RR (95% C.I.)^#^	N (%)	RR (95% C.I.)^#^	N (%)	RR (95% C.I.)^#^
**Wealth Quintile**								
**Q1 - Poorest** (n = 21)	20 (95.2%)	1.6 (1.2–2.0)	12 (57.1%)	3.9 (1.7–8.9)	10 (47.6%)	4.9 (1.7–13.7)	5 (23.8%)	4.9 (1.0–23.2)
**Q2** (n = 38)	35 (92.1%)	1.5 (1.2–2.0)	14 (36.8%)	2.5 (1.1–5.9)	12 (31.6%)	3.2 (1.1–9.2)	4 (10.5%)	2.2 (0.4–11.2)
**Q3** (n = 50)	42 (84.0%)	1.4 (1.1–1.8)	16 (32.0%)	2.2 (0.9–5.1)	7 (14.0%)	1.4 (0.1–2.6)	5 (10.0%)	2.1 (0.4–10.1)
**Q4** (n = 50)	38 (76.0%)	1.2 (0.9–1.7)	4 (8.0%)	0.5 (0.2–1.8)	3 (6.0%)	0.6 (0.1–2.6)	1 (2.0%)	0.4 (0.0–4.4)
Q5*- Wealthiest (n = 41)	25 (61.0%)	Ref	6 (14.6%)	Ref	4 (9.8%)	Ref	2 (4.9%)	Ref
**Geographical residence**								
Urban* (n = 29)	17 (58.6%)	Ref	4 (13.8%)	Ref	3 (10.3%)	Ref	2 (6.9%)	Ref
**Rural** (n = 171)	143 (83.6%)	1.4 (1.0–1.9)	48 (28.1%)	2.0 (0.8–5.2)	33 (19.3%)	1.9 (0.6–5.7)	15 (8.8%)	1.3 (0.3–5.3)
**Maternal education**								
**None/ early** **childhood** (n = 53)	44 (97.8%)	1.6 (1.3–1.8)	20 (44.4%)	2.8 (1.6–5.2)	15 (33.3%)	4.6 (1.9–11.1)	9 (20.0%)	8.3 (1.9–36.9)
**Primary** (n = 83)	55 (88.7%)	1.4 (1.2–1.7)	17 (27.4%)	1.7 (0.9–3.3)	14 (22.6%)	3.1 (1.3–7.7)	5 (8.1%)	3.4 (0.7–16.8)
**Secondary or higher** (n = 248)	52 (62.7%)	Ref	13 (15.7%)	Ref	6 (7.2%)	Ref	2 (2.4%)	Ref
**Ethno-linguistic group**								
Lao-Tai* (n = 168)	129 (76.8%)	Ref	37 (22.0%)	Ref	23 (13.7%)	Ref	11 (6.6%)	Ref
**Mon-Khmer** (n = 29)	28 (96.6%)	1.3 (1.1–1.4)	14 (48.3%)	2.2 (1.4–3.5)	12 (41.4%)	3.0 (1.7–5.4)	6 (20.7%)	3.2 (1.3–7.9)
**Hmong-Mien** (n = 0)	N/A	N/A	N/A	N/A	N/A	N/A	N/A	N/A
**Chinese-Tibetan** (n = -0)	N/A	N/A	N/A	N/A	N/A	N/A	N/A	N/A
**Other** (n = 3)	3 (100%)	1.3 (1.2–1.4)	1 (33.3%)	1.5 (0.3–7.7)	1 (33.3%)	2.4 (0.5–12.7)	0	–

CHE = catastrophic health expenditure; HE = household expenditure; CTP = capacity to pay.

* Reference group.

# Due to non-convergence, RR estimated using Poisson regression with robust standard errors.

Risk ratios were also calculated across demographic groups for impoverishment rates. At SPH, higher impoverishment rates based on the national poverty line, were seen in the poorest (Q1) versus wealthiest quintile (Q5) (RR 2.6, 95% CI 1.3 – 5.4), households with no formal maternal education compared to secondary education or higher (RR 7.4, 95% CI 1.1 – 51.9) and the Mon-Khmer versus Lao-Tai ethnic groups (RR 2.4, 95% C.I. 1.4 – 4.1). At NCH, findings were limited due to overall low numbers of impoverishment. *Details of these results are included in S5 Table and S6 Table.*

### Financial coping strategies

The OOP costs were calculated after health insurance rebates (where applicable) were accounted for. Further financial coping strategies that households reported to help cover OOP costs related to the severe illness at discharge from hospital included: use of savings (overall 394/400 = 98.5%), borrowing money from relatives or friends (NCH 17.9%, SPH 14.5%), reducing household expenses (NCH 3.1%, SPH 0%), selling assets (two households), borrowing money from the bank (two households) and delaying plans (one household). *Refer to* *S7 Table* *for a summary of coping strategies reported at each study visit.*

## Discussion

Our study aimed to describe the financial burden on households with sick children requiring inpatient care in Lao PDR, in the context of NHI. We found that households paid USD92.4 at a secondary level, provincial hospital with NHI, and USD290.6 at a tertiary level, central hospital without NHI, in median total OOP costs related to an acute episode of severe illness. CHE (direct OOP costs >10% of annual household expenditures) rates at both hospitals (NCH 22.5%, SPH 18.0%) were higher than globally reported CHE rates of 13.5% [11]. Impoverishment rates in our study as a result of direct OOP costs were higher at SPH (10.2% - 16.0%) than NCH (0.5% - 1.5%). This is in comparison to global impoverishment rates of 4.4% [11].

Higher total OOP costs at NCH households were likely due to additional factors unrelated to NHI. Tertiary hospitals are generally more expensive compared to secondary level hospitals. They have higher bed costs, treat more complex cases, with access to more advanced equipment and diagnostics with higher associated costs [32,33]. NCH patients had a higher ICU admission rate with longer hospital stays than SPH patients, resulting in higher direct medical costs. Reasons for hospital admission also varied between the hospitals likely affecting OOP costs. The main primary diagnosis at NCH was pneumonia (62.3%), compared to SPH with pneumonia in 35.5% of participants followed by upper respiratory tract infections in 21.5%. Pneumonia is a more severe condition than upper respiratory tract infections, and requires treatment such as antibiotics and in more severe cases oxygen supplementation and respiratory support [23]. Although our study did not include an analysis of the investigations and treatment received in hospital, differences in diagnoses with more severe conditions at NCH likely led to differences in management and hence OOP costs. Furthermore, primary income earners at NCH had regular salaries and higher estimated incomes than at SPH, where household heads were predominantly farmers. This resulted in higher indirect costs at NCH from productivity loss.

Despite NCH households having higher OOP costs, rates of CHE were similar and impoverishment rates were conversely higher at SPH households. CHE and impoverishment are calculated relative to estimated household expenses, which reflect local living costs and spending patterns. Economic analyses of household data from the Lao Expenditure and Consumption Survey has shown that the wealthiest quintile consume seven times more on average per capita than the poorest quintile, and rural areas spend more of their household budget on food compared to urban areas (68% versus 53%) [17]. In our study NCH compared with SPH had a much higher proportion of households from the wealthiest quintile (79% versus 20.5%) and urban areas (87% versus 14.5%), with higher household expenses. The lower OOP costs but inversely higher CHE and impoverishment rates at SPH may therefore be explained in part by their lower income and hence household expenses. Disparities in expendable income and household spending patterns across subpopulations suggest that health related financial hardship is not just dependent on OOP costs due to the medical event and hence cannot be fully mitigated by the NHI alone.

CHE and impoverishment rates found in our study were high compared with other South-East Asian countries. A study by Wang et al, conducted in eight countries in South-East Asia (Bangladesh, Bhutan, India, Maldives, Nepal, Sri Lanka, Thailand and Timor-Leste), found CHE rates (direct medical OOP costs >10% of annual household expenditures) ranging from 1.9% (Thailand) to 19.9% (Maldives). Impoverishment rates using the international poverty line ranged from 0% (Thailand) to 4.2% (India) [6]. Based on the same thresholds, our study had a CHE rate of 13.3% and impoverishment rates of 2.7% - 5.9%. Thailand, which neighbours Lao PDR, is seen as an example where government investment in health and a well implemented NHI scheme has translated to lower financial hardship rates on par with high income countries [34]. Lao PDR is yet to emulate similar results from their NHI scheme, with challenges of insufficient budget, low revenues from copayments, operational issues and continuing government health expenditures negatively impacted by the COVID-19 pandemic [21].

Large equity disparities between demographic subgroups were found in our study with higher financial hardship rates in the poorest households, rural areas, households with low maternal education and ethnic minority groups. Similar trends of inequity have been shown in other LMIC studies assessing the financial burden of health care for sick children. A Kenyan study found CHE occurring almost four times as often in lower socio-economic households than higher ones [35]. A study of sick children in Bangladesh showed that households with mothers who had no formal education needed external financial assistance to fund OOP costs three times more often than households with maternal secondary education levels or higher [36]. Inequitable financial hardship however, are not limited to LMICs. A systematic review on the determinants of CHE found similar socio-economic disparities in CHE rates to our study, across low, middle and high income countries, with health insurance associated with lower CHE rates in only 9/17 studies [37].

The NHI scheme aims to improve equitable access to health care and reduce unmet medical needs in Lao PDR. Since its introduction, there has been some evidence that the NHI has increased health service utilisation for the poorest households [18,38]. This was also indicated in our study with participants from SPH where the NHI is available, being relatively equally distributed across wealth quintiles, compared to NCH where participants were almost exclusively from the two wealthiest quintiles. Although this reflects similar wealth distribution to national survey data where 90% of households in the Capital City are from the two wealthiest quintiles, our findings suggest that the small percentage of poorer households in the Capital City with severely sick children either sought care from other likely lower level, cheaper health centres or did not receive health care at all. Further evaluation is needed to determine whether the NHI being excluded from the Capital City is resulting in low health care access from poorer households to ensure these vulnerable populations are not overlooked in any health programs and policy reforms.

Our study also had a small proportion of participants from ethnic minority groups at both hospitals with 85.3% from the Lao-Tai ethno-linguistic group, compared to a national average of 62.4% [15]. This may be due to our limited sample, but it may also reflect cultural barriers for ethnic minority groups accessing health care. Other studies from neighbouring Asian countries have shown that ethnic minorities face higher vulnerabilities that hinder adequate health care access such as being from poorer households, living in remote areas with limited transportation, lower health literacy, and culturally inappropriate practices from medical personnel [39,40]. The NHI aims to mitigate some of these barriers by providing additional incentives to free health care such as travel and food vouchers for the poor and remote populations. There is a dearth in the literature on the impact of health insurance on ethnic minority groups in LMICs with conflicting evidence as to whether social protection schemes improve or worsen healthcare access for ethnic minorities and displaced populations [41–43]. More research extending to other provinces where NHI is available, is required to add to the limited literature on the impact of NHI on equitable access to care, especially for ethnic minority groups.

Definitions for financial protection are inconsistent, requiring careful interpretation and comparison of results.The SDGs use direct (medical and non-medical) OOP expenses, to calculate CHE and impoverishment rates which ignores the impact of indirect costs on households. In our study we calculated OOP costs using both direct and indirect expenses, recognising the potential significant financial burden for caregivers foregoing income to care for their sick child [2]. We found that indirect costs increased the total OOP expenses by 50% – 60%. Similar findings were seen in a study in Malaysia evaluating the financial impact of hospitalised childhood gastroenteritis which showed indirect costs contributed 44% – 74% to total OOP costs and increased CHE rates at one of the hospitals from one third to 88% of households, compared with direct OOP costs alone [44]. A further hidden impact of indirect costs are the missed healthcare opportunities where families decide to not seek care or leave hospital early due to inaffordability with likely negative health impacts. In our study, 26/400 (6.6%) patients were discharged early without completing medical treatment, four of whom died during the follow-up period.

There are also variable thresholds to define CHE and impoverishment. Previous studies have shown that using the household “capacity-to-pay” rather than household income as the denominator to calculate CHE yields more accurate prevalence in poorer households, as they spend a larger proportion of their income on subsistence items [2,4,5,18,25–27,45]. In Lao PDR where much of the country relies on informal economic activity and subsistence farming, our study also showed larger equity gaps in CHE rates when using capacity-to-pay compared with using household expenditure, between the poorest and wealthiest quintiles, between rural and urban areas and between those with no education and higher education levels. In our study, poverty headcounts using the national poverty line showed larger inequity gaps across all subpopulation groups than when using the international poverty line. Although the international poverty line is useful for comparing extreme poverty between countries, the Lao national poverty line is a better reflection of local living standards and more accurately depicts poverty across populations groups within the country [17]. Despite the variability and need for careful interpretation, using multiple thresholds allow for comparisons between and within countries, with data from this study making valuable contribution in global monitoring efforts towards achieving the SDGs.

Current metrics used to calculate and identify rates of financial hardship however, do not consider how households pay for OOP health expenses, or “financial coping mechanisms”. In LMICs, including Lao PDR, it is common for families to borrow money, use savings or sell assets and land to pay for OOP health expenditures, with rural households and those of lower income more likely to use multiple coping strategies and/or borrow larger amounts of money. [5,46–48]. The impact of financial coping mechanisms can continue well past the physical health outcomes. Households may struggle to repay loans and resort to cutting-back on subsistence spending, exposing them to further stressors [47,48]. Our study showed there was lingering financial burden two months after being discharged from hospital, with almost half of the households reporting still using savings to pay for OOP expenses and around 3% of households yet to pay back money borrowed from relatives or friends. There should be consideration for the hidden impact of household financial coping mechanisms in government policy and interventions towards financial protection.

Our study had several limitations. Data were collected primarily from self-report, potentially leading to recall bias. We tried to reduce this by limiting the recall period to two weeks and providing diaries for households to document expenses inbetween study visits. Our study only included patients from two hospitals which may not be representative of the entire population of Lao PDR. We however, endeavoured to recruit a broad demographic by including hospitals from two different regions of Lao PDR (central and south). The hospitals selected by our Lao investigators, with extensive knowledge of the local health service landscape, were deemed to have the largest catchment in these regions, and wide demographic range. Non Lao speaking parents were excluded from the study potentially omitting disadvantaged ethnic minority groups and biasing the cohort. Unequal wealth distribution at NCH prevented assessment of health care costs on poorer groups in the Capital City, likely underestimating CHE and impoverishment rates. However, this may be due to the inaffordability of health care costs for poorer households in the Capital City, rather than a limitation in our study methodology. Further study is required across regions and levels of healthcare facilities, to understand barriers to accessing care and how the NHI scheme has impacted this, with focus on highly vulnerable groups not eligible for NHI being considered in any restructuring of the NHI scheme.

## Conclusion

Severe childhood illnesses can result in high OOP costs leading to financial hardship for households. The Lao NHI scheme aims to reduce health related costs for households and decrease health inequities. Our study shows that while direct medical OOP expenses for hospitalised children are relatively low where NHI is available, CHE and impoverishment rates remain high with inequitable distribution towards the poor, those living in rural areas, with lower maternal education levels and ethnic minority groups. Further investment in interventions beyond the NHI is needed, including evaluation of their impact, to prevent severe childhood illnesses and reduce non-medical related costs of illnesses that contribute to financial hardship. Additional study is required to explore underlying causes of disparities among disadvantaged groups including those in the Capital City not eligible for NHI. These populations need further financial protection and support accessing health services to reduce health inequities and overall financial burden on households and in Lao PDR.

## Supporting information

S1 Checklist STROBE Statement – checklist of items that should be included in reports of cohort studies [22]. (DOCX)

S2 Checklist Inclusivity in global research questionnaire. (DOCX)

S1 Table Mean out-of-pocket costs (in USD) associated with severe illness, by hospital. USD = United States Dollar, OOP = out-of-pocket. *Complete case analysis (i.e., Data were available for all study visits).
(DOCX)

S2 Table Itemised costs contributing to total out-of-pocket costs (in USD) associated with severe illness by hospital. USD = United States Dollar. *Visit 1 = at enrolment during hospital admission; Visit 2 = at hospital discharge; Visit 3 = 2 weeks post hospital discharge; Visit 4 = 2 months post hospital discharge.
(DOCX)

S3 Table Catastrophic health expenditure rates associated with severe illness, by hospital. HE = household expenditures; CTP = capacity to pay.
(DOCX)

S4 Table Impoverishment rates based on direct (medical & non-medical) OOP costs associated with severe illness, by hospital. USD = United States Dollar; LAK = Lao Kip.
(DOCX)

S5 Table Impoverishment rates based on direct (medical + non-medical) costs, by demographics at National Children’s Hospital. USD = United States Dollar; LAK = Lao Kip. *Reference group. ^#^Due to non-convergence, RR estimated using Poisson regression with robust standard errors.
(DOCX)

S6 Table Impoverishment rates based on direct (medical + non-medical) costs, by demographics at Salavan Provincial Hospital. USD = United States Dollar; LAK = Lao Kip. *Reference group. ^#^Due to non-convergence, RR estimated using Poisson regression with robust standard errors.
(DOCX)

S7 Table Reported financial coping strategies to pay for out-of-pocket costs associated with severe illness by hospital. *Visit 1 = at enrolment during hospital admission; Visit 2 = at hospital discharge; Visit 3 = 2 weeks post hospital discharge; Visit 4 = 2 months post hospital discharge.
(DOCX)
