# Supplementary material for: Financial burden of severe childhood illness on households in Lao People’s Democratic Republic: A prospective cohort study
Source: PLOS Glob Public Health. 2026 Feb 20;6(2):e0004783. doi: 10.1371/journal.pgph.0004783 (PMC12923058; doi:10.1371/journal.pgph.0004783)
Supplement: S2 Checklist — (DOCX) [file pgph.0004783.s002.docx]

Inclusivity in global research

PLOS’ policy on inclusivity in global research aims to improve transparency in the reporting of research performed outside of researchers’ own country or community and ensures that PLOS publications reporting global research adhere to high standards for research ethics and authorship. Authors of relevant research articles may be asked to complete the questionnaire below, which outlines ethical, cultural, and scientific considerations specific to inclusivity in global research. This questionnaire may be requested when researchers have travelled to a different country to conduct research, if research uses samples collected in another country, research with Indigenous populations or their lands, or if research is on cultural artefacts. Researchers travelling to another country solely to use laboratory equipment will not normally be required to complete the questionnaire. However, the questionnaire can be requested at the journal’s discretion for any submission – if you have been requested to complete this questionnaire by the PLOS journal you submitted to, please do so.

Please complete the questionnaire below and include this as a Supporting Information file with your manuscript. Note that if your paper is accepted for publication, this checklist will be published with your article in the supporting information files. Please ensure that you reference the checklist in the main body of your manuscript. We suggest adding a subsection ‘Inclusivity in global research’ to your Methods section and adding the following sentence: “Additional information regarding the ethical, cultural, and scientific considerations specific to inclusivity in global research is included in the Supporting Information (SX Checklist)”

The questions have been designed to be applicable to a wide range of study types, and there are subsections for both human subjects research and non-human subjects research. If any of the questions are not relevant to your research please mark them as “N/A” as appropriate.

**Ethical considerations, permits and authorship**

*This section is applicable to all research types.*

Provide details as to who granted permissions and/or consent for the study to take place in the Methods section of your manuscript. This should include the names of **all** ethics boards, governmental organizations, community leaders or other bodies that provided approval for the study. If individuals provided approval refer to these people by their role or title but do not list their name(s).

Reported on page number: 9

The study was conducted according to the protocol approved by The Royal Children’s Hospital Human Research Ethics Committee (HREC 81864/RCHM-2022), the University of Oxford Tropical Research Ethics Committee (552-22), and the Lao PDR Ministry of Health, National Ethics Committee for Health Research (2022.52).

If there were any deviations from the study protocol after approval was obtained please provide details of these changes in the Methods section of your manuscript.

Reported on page number: Not applicable.

There were no deviations from the study protocol.

Did this study involve local collaborators that are residents of the country where the research was conducted or members of the community studied? If you do not have any authors from said communities, please provide an explanation for this below.

Yes. The study involved local collaborators from Lao PDR in the study design, protocol development, study procedures as well as local project management. The study was conducted in collaboration with the locally-based Lao-Oxford-Mahosot Hospital-Wellcome Trust Research Unit (LOMWRU). The study protocol was developed in consultation with local site principal investigator (MM) who has had extensive experience in conducting field clinical research in Lao PDR. All study visits were conducted by local research staff with daily oversite from a local study coordinator.

Everyone listed as an author should meet PLOS’ criteria for authorship and all individuals who meet these criteria should be included in the author byline, rather than the acknowledgements. For further information please see the journal’s Authorship Policy.

**Human subjects research (e.g. health research, medical research, cross-cultural psychology)**

Did you obtain written informed consent from a representative of the local community or region before the research took place? How did you establish who speaks for the community? Details of written informed consent obtained from study participants should be reported separately in the Methods section of your manuscript.

Ethical approval of the study protocol was obtained from the Lao PDR National Ethics Committee for Health Research (NECHR) prior to commencement of they study. This was to ensure that our study complied with local and international ethical standards, as well as reflect appropriate activities for the Lao setting. The site principal investigator (MM) and local study coordinator (LB) collaborated and obtained consent from the Directors and Head of Units of the hospital sites to ensure feasibility of study and cultural appropriateness. Written informed consent were obtained from all study participants (page 5 in Methods section of manuscript).

How did members of the local community provide input on the aims of the research investigation, its methodology, and its anticipated outcome(s)?

The aims and design of the study were developed in collaboration with our Lao investigators. This involved multiple team meetings and discussion to ensure our study aims and direction addressed local health priorities in Lao PDR, as well as being feasible to be implemented with local resources. The Site principal investigator (MM) is Head of Field Research at LOMWRU and has extensive experience in clinical research locally. The anticipated outcomes were deemed of high priority and interest to the local government.

When engaging with the local community, how did you ensure that the informed consent documents and other materials could be understood by local stakeholders?

The study protocol, consent and participant information forms, and study tools were all developed in conjunction with our Lao collaborators. All consent forms, patient information sheets and questionnaires were translated by our local investigators into the Lao language and back-translated into English. This was to ensure accurate translation. Questionnaires were tested in Lao with a small sample prior to commencing the study, to ensure appropriate use of lay-language and to provide feedback for any adjustments required.

Will the findings of the research be made available in an understandable format to stakeholders in the community where the study was conducted (e.g. via a presentation, summary report, copies of publications, etc.)? Please provide details of how this will be achieved.

The findings will be made available through multiple methods. Oral presentation of findings will be presented at multiple stakeholder meetings as well as international conferences in Australia and in Lao PDR. These will be attended by the local stakeholders and include the research teams, staff from the hospital sites, local government and non-government agencies. A summary report will be provided to local stakeholders. Findings will be published in a peer-reviewed free-access journal. Research team members have had substantial input to the peer-reviewed publication and named as co-authors.

**Non-human subjects research using specimens/ animals collected as part of the study, or those housed in archival collections. Examples include archaeology, paleontology, botany and zoology.**

Did the permission you obtained from a local authority to perform the study include an agreement on access to outputs and benefit sharing? This may include procedures to enable fair distribution of the benefits and resources arising from the research performed. Please include any details of Prior Informed Consent and Benefit Sharing Agreements obtained. These may be required by field-specific regulations, for example the Convention on Biological Diversity (CBD) and the associated Nagoya Protocol.

Not applicable

If the material used in your study was imported, please A) provide the year it was imported and B) indicate whether permits were obtained to import/export the materials used, C) provide details of any permits obtained. If this information is not available, please indicate this.

Not applicable

If you used archival specimens, please state how the material used in your study was acquired by the institute it is held in and provide details of any permits obtained for the original excavations/ sample collection. If this information is not available, please indicate this.

Not applicable

How was the potential cultural significance of the materials collected in your study to local communities considered in your research design? Were Indigenous peoples and/or local researchers and institutions involved with archaeological excavations / collection of specimens? If so, please provide a description of their involvement.

Not applicable

If your manuscript includes photographs of human remains please indicate whether authors obtained permission from descendants or affiliated cultural communities to do so.

Not applicable
