# Supplementary material for: Financial burden of severe childhood illness on households in Lao People’s Democratic Republic: A prospective cohort study
Source: PLOS Glob Public Health. 2026 Feb 20;6(2):e0004783. doi: 10.1371/journal.pgph.0004783 (PMC12923058; doi:10.1371/journal.pgph.0004783)
Supplement: S1 Table — USD = United States Dollar, OOP = out-of-pocket. *Complete case analysis (i.e., Data were available for all study visits). (DOCX) [file pgph.0004783.s003.docx]

**S1 Table: Mean out-of-pocket costs (in USD) associated with severe illness, by hospital**

|  | **National Children’s Hospital**  N=185* | **Salavan Provincial Hospital**  N=186* |
| --- | --- | --- |
| **Direct medical costs, mean (SD))** | 206.2 (329.6) | 28.0 (189.7) |
| **Direct non-medical OOP costs, mean (SD)** | 74.4 (55.1) | 72.5 (71.2) |
| **Indirect OOP costs,**  **mean (SD)** | 113.6 (130.8) | 52.2 (91.2) |
| **Total OOP (direct medical + direct non-medical costs, mean (SD))** | 280.6 (375.0) | 100.6 (238.6) |
| **Total OOP (all direct + indirect costs, mean (SD))** | 394.2 (442.2) | 152.8 (263.5) |

USD = United States Dollar, OOP = out-of-pocket

*Complete case analysis (ie. Data available for all study visits)
