# Supplementary material for: Financial burden of severe childhood illness on households in Lao People’s Democratic Republic: A prospective cohort study
Source: PLOS Glob Public Health. 2026 Feb 20;6(2):e0004783. doi: 10.1371/journal.pgph.0004783 (PMC12923058; doi:10.1371/journal.pgph.0004783)
Supplement: S2 Table — USD = United States Dollar. *Visit 1 = at enrolment during hospital admission; Visit 2 = at hospital discharge; Visit 3 = 2 weeks post hospital discharge; Visit 4 = 2 months post hospital discharge. (DOCX) [file pgph.0004783.s004.docx]

**S2 Table: Itemised costs contributing to total out-of-pocket costs (in USD) associated with severe illness by hospital**

|  | **National Children’s Hospital (N=200)** | | | | | **Salavan Provincial Hospital (N=200)** | | | | |
| --- | --- | --- | --- | --- | --- | --- | --- | --- | --- | --- |
|  | Visit 1* | Visit 2* | Visit 3* | Visit 4* | **All visits** | Visit 1* | Visit 2* | Visit 3* | Visit 4* | **All visits** |
| **Direct medical costs in USD (mean)** | | | | | | | | | | |
| **Hospital bed fees** | 0 | 24.3 | 2.0 | 1.2 | **27.5** | 0 | 3.3 | 2.6 | 0.2 | **6.1** |
| **Consultation fees** | 0.1 | 0 | 0.5 | 0.3 | **0.9** | 0 | 0 | 0.9 | 0 | **0.9** |
| **Medicines** | 10.8 | 118.4 | 7.4 | 9.2 | **145.8** | 4.3 | 29.9 | 8.6 | 2.9 | **45.7** |
| **Medical investigations** | 4.4 | 32.9 | 2.0 | 3.8 | **43.1** | 0.8 | 10.1 | 4.1 | 0.4 | **15.4** |
| **Transfer fees** | 0.6 | 0 | 0 | 0 | **0.6** | 0.1 | 0 | 0 | 0 | **0.1** |
| **Other direct medical costs** | 0.3 | 1.1 | 0.2 | 9.3 | **10.9** | 0.5 | 1.5 | 3.1 | 0.3 | **5.4** |
| **Direct non-medical costs in usd (mean)** | | | | | | | | | | |
| **Travel costs** | 6.9 | 13.0 | 1.6 | 3.9 | **25.4** | 5.4 | 13.7 | 4.6 | 2.5 | **26.2** |
| **Childcare costs for other children (not usually paid)** | 0 | 0 | 0 | 0 | **0** | 0 | 0.1 | 0 | 0 | **0.1** |
| **Daily living expenses** | 0 | 47.1 | 0 | 0 | **47.1** | 0 | 45.3 | 0 | 0 | **45.3** |
| **Accommodation expenses** | 0 | 0.4 | 0 | 0 | **0.4** | 0 | 0 | 0 | 0 | **0** |
| **Indirect costs in usd (mean)** | | | | | | | | | | |
| **Income loss for primary income earner** | 21.3 | 37.4 | 5.3 | 6.6 | **70.6** | 14.5 | 11.9 | 3.3 | 1.3 | **31** |
| **Income loss for primary caregiver** | 13.5 | 20.4 | 4.4 | 4.1 | **42.4** | 8.4 | 8.0 | 2.1 | 1.1 | **19.6** |

USD = United States Dollar

*Visit 1 = at enrolment during hospital admission; Visit 2 = at hospital discharge; Visit 3 = 2 weeks post hospital discharge; Visit 4 = 2 months post hospital discharge
