# Supplementary material for: Financial burden of severe childhood illness on households in Lao People’s Democratic Republic: A prospective cohort study
Source: PLOS Glob Public Health. 2026 Feb 20;6(2):e0004783. doi: 10.1371/journal.pgph.0004783 (PMC12923058; doi:10.1371/journal.pgph.0004783)
Supplement: S3 Table — HE = household expenditures; CTP = capacity to pay. (DOCX) [file pgph.0004783.s005.docx]

**S3 Table: Catastrophic health expenditure rates associated with severe illness, by hospital**

|  | **National Children’s Hospital**  N=200 | **Salavan Provincial Hospital**  N=200 |
| --- | --- | --- |
| **Threshold: 10% of two-monthly HE**  **N (%) (95% C.I.)** | | |
| **Direct medical costs** | 142 (71.0%)  (64.7 - 77.3) | 31 (15.5%)  (10.5 - 20.5) |
| **All direct costs** | 176 (88.0%)  (83.5 - 92.5) | 160 (80.0%)  (74.5 - 85.5) |
| **Direct + indirect costs** | 194 (97.0%)  (94.6 - 99.4) | 185 (92.5%)  (88.8 - 96.2) |
| **Threshold: 40% of two-monthly CTP**  **N (%) (95% C.I.)** | | |
| **Direct medical costs** | 62 (31.0%)  (24.6 - 37.4) | 19 (9.5%)  (5.4 - 13.6) |
| **All direct costs** | 82 (41.0%)  (34.2 - 47.8) | 52 (26.0%)  (19.9 - 32.1) |
| **Direct + indirect costs** | 113 (56.5%)  (49.6 - 63.4) | 88 (44.0%)  (37.1 - 50.9) |
| **Threshold: 10% of annual HE**  **N (%) (95% C.I.)** | | |
| **Direct medical costs** | 37 (18.5%)  (13.1 - 23.9) | 16 (8.0%)  (4.2 - 11.7) |
| **All direct costs** | 45 (22.5%)  (16.7 - 28.3) | 36 (18.0%)  (12.7 - 23.3) |
| **Direct + indirect costs** | 69 (34.5%)  (27.9 - 41.1) | 52 (26.0%)  (19.9 - 32.1) |
| **Threshold: 40% of annual CTP**  **N (%) (95% C.I.)** | | |
| **Direct medical costs** | 17 (8.5%)  (5.0 - 13.3) | 15 (7.5%)  (4.3 - 12.1) |
| **All direct costs** | 18 (9.0%)  (5.0 - 13.0) | 17 (8.5%)  (4.6 - 12.4) |
| **Direct + indirect costs** | 19 (9.5%)  (5.4 - 13.6) | 19 (9.5%)  (5.4 - 13.6) |

HE = household expenditures; CTP = capacity to pay
