# Supplementary material for: Financial burden of severe childhood illness on households in Lao People’s Democratic Republic: A prospective cohort study
Source: PLOS Glob Public Health. 2026 Feb 20;6(2):e0004783. doi: 10.1371/journal.pgph.0004783 (PMC12923058; doi:10.1371/journal.pgph.0004783)
Supplement: S4 Table — USD = United States Dollar; LAK = Lao Kip. (DOCX) [file pgph.0004783.s006.docx]

**S4 Table: Impoverishment rates based on direct (medical & non-medical) OOP costs associated with severe illness, by hospital**

|  | **National Children’s Hospital**  N=200 | **Salavan Provincial Hospital**  N=200 |
| --- | --- | --- |
| **Impoverishment at baseline (time of recruitment)** | | |
| **Threshold: International poverty line $USD 2.15/person/day at 2017 PPP** | | |
| **Poverty headcount**  **N (%) (95% C.I.)** | 2 (1.0%)  (0.4 - 2.4) | 32 (16.0%)  (10.9 - 21.1) |
| **Households pushed below poverty line N (%)** | 2 (1.0%) | 12 (6.0%) |
| **Threshold: Laos national poverty line LAK 280,910/person/month ($USD 2.00/person/month)** | | |
| **Poverty headcount**  **N (%) (95% C.I.)** | 5 (2.5%)  (0.3 - 4.7) | 69 (34.5%)  (27.9 - 41.1) |
| **Households pushed below poverty line N (%)** | 4 (2.0%) | 10 (5.0%) |
| **Impoverishment at 2 weeks after hospital discharge** | | |
| **Threshold: International poverty line $USD 2.15/person/day at 2017 PPP** | | |
| **Poverty headcount**  **N (%) (95% C.I.)** | 3 (1.5%)  (0.0 - 3.3) | 26 (13.0%)  (8.3 - 17.7) |
| **Households pushed below poverty line N (%)** | 3 (1.5%) | 20 (10.0%) |
| **Threshold: Laos national poverty line LAK 280,910/person/month ($USD 2.00/person/month)** | | |
| **Poverty headcount**  **N (%) (95% C.I.)** | 5 (2.6%)  (0.3 - 4.8) | 57 (28.5%)  (22.3 - 34.8) |
| **Households pushed below poverty line N (%)** | 5 (2.6%) | 35 (17.5%) |
| **Impoverishment at 2 months after hospital discharge** | | |
| **Threshold: International poverty line $USD 2.15/person/day at 2017 PPP** | | |
| **Poverty headcount**  **N (%) (95% C.I.)** | 1 (0.5%)  (0.0 - 3.0) | 19 (10.2%)  (6.2 - 15.4) |
| **Households pushed below poverty line N (%)** | 1 (0.5%) | 9 (4.8%) |
| **Threshold: Laos national poverty line LAK 280,910/person/month ($USD 2.00/person/month)** | | |
| **Poverty headcount**  **N (%) (95% C.I.)** | 2 (1.1%)  (0.1 - 3.8) | 45 (24.1%)  (18.1 - 30.8) |
| **Households pushed below poverty line N (%)** | 2 (1.1%) | 28 (15.0%) |

USD = United States Dollar; LAK = Lao Kip
