# Supplementary material for: Financial burden of severe childhood illness on households in Lao People’s Democratic Republic: A prospective cohort study
Source: PLOS Glob Public Health. 2026 Feb 20;6(2):e0004783. doi: 10.1371/journal.pgph.0004783 (PMC12923058; doi:10.1371/journal.pgph.0004783)
Supplement: S5 Table — USD = United States Dollar; LAK = Lao Kip. *Reference group. #Due to non-convergence, RR estimated using Poisson regression with robust standard errors. (DOCX) [file pgph.0004783.s007.docx]

**S5 Table. Impoverishment rates based on direct (medical + non-medical) costs, by demographics at National Children’s Hospital**

| **Impoverishment threshold** | **International poverty line $USD 2.15/person/day at 2017 PPP** | | | | **Laos national poverty line LAK 280,910/person/month**  **($USD 22.00/person/month)** | | | |
| --- | --- | --- | --- | --- | --- | --- | --- | --- |
|  | **At baseline** | | **At 2 months post-discharge** | | **At baseline** | | **At 2 months post-discharge** | |
|  | N (%) | RR (95% C.I.)^#^ | N (%) | RR (95% C.I.)^#^ | N (%) | RR (95% C.I.)^#^ | N (%) | RR (95% C.I.)^#^ |
| **Wealth Quintile** | | | | | | | | |
| **Q1 - Poorest** (n=0) | N/A | N/A | N/A | N/A | N/A | N/A | N/A | N/A |
| **Q2** (n=3) | 0 | - | 0 | - | 0 | - | 0 | - |
| **Q3** (n=2) | 1 (50.0%) | 79.0 (7.2 – 866.8) | 0 | - | 1 (50.0%) | 79.0 (7.2 – 866.8) | 0 | - |
| **Q4** (n=37) | 0 | - | 1 (2.8%) | - | 3 (8.1%) | 12.8 (1.4 – 119.7) | 1 (2.8%) | 4.14 (0.3 – 64.6) |
| **Q5*- Wealthiest** (n=158) | 1 (0.6%) | Ref | 0 | Ref | 1 (0.6%) | Ref | 1 (0.6%) | Ref |
| **Geographical residence** | | | | | | | | |
| **Urban*** (n=174) | 1 (0.6%) | Ref | 1 (0.6%) | Ref | 3 (1.7%) | Ref | 2 (1.2%) | Ref |
| **Rural** (n=26) | 1 (3.9%) | 6.7 (0.4 – 104.5) | 0 | - | 2 (7.7%) | 4.5 (0.8 – 25.6) | 0 | - |
| **Maternal education** | | | | | | | | |
| **None / early**  **childhood** (n=8) | 0 | - | 0 | - | 0 | - | 0 | - |
| **Primary** (n=21) | 0 | - | 0 | - | 1 (4.8%) | 2.0 (0.2 – 16.8) | 0 | - |
| **Secondary or higher** (n=165) | 2 (1.2%) | Ref | 1 (0.6%) | Ref | 4 (2.4%) | Ref | 1 (0.6%) | Ref |
| **Ethno-linguistic Group** | | | | | | | | |
| **Lao-Tai*** (n=173) | 0 | - | 0 | - | 2 (1.2%) | Ref | 1 (0.6%) | Ref |
| **Mon-Khmer** (n=6) | 0 | - | 0 | - | 0 | - | 0 | - |
| **Hmong-Mien** (n=19) | 2 (10.5%) | Ref | 1 (6.3%) | Ref | 3 (15.8%) | 13.7 (2.4 – 77.0) | 1 (6.3%) | 10.2 (0.7 – 156.4) |
| **Chinese-Tibetan** (n=1) | 0 | - | 0 | - | 0 | - | 0 | - |
| **Other** (n=1) | 0 | - | 0 | - | 0 | - | 0 | - |

USD = United States Dollar; LAK = Lao Kip

*Reference group

^#^Due to non-convergence, RR estimated using Poisson regression with robust standard errors
