# Supplementary material for: Financial burden of severe childhood illness on households in Lao People’s Democratic Republic: A prospective cohort study
Source: PLOS Glob Public Health. 2026 Feb 20;6(2):e0004783. doi: 10.1371/journal.pgph.0004783 (PMC12923058; doi:10.1371/journal.pgph.0004783)
Supplement: S6 Table — USD = United States Dollar; LAK = Lao Kip. *Reference group. #Due to non-convergence, RR estimated using Poisson regression with robust standard errors. (DOCX) [file pgph.0004783.s008.docx]

**S6 Table. Impoverishment rates based on direct (medical + non-medical) costs, by demographics at Salavan Provincial Hospital**

| **Impoverishment threshold** | **International poverty line $USD 2.15/person/day at 2017 PPP** | | | | **Laos national poverty line LAK 280,910/person/month**  **($USD 22.00/person/month)** | | | |
| --- | --- | --- | --- | --- | --- | --- | --- | --- |
|  | **At baseline** | | **At 2 months post-discharge** | | **At baseline** | | **At 2 months post-discharge** | |
|  | N (%) | RR (95% C.I.)^#^ | N (%) | RR (95% C.I.)^#^ | N (%) | RR (95% C.I.)^#^ | N (%) | RR (95% C.I.)^#^ |
| **Wealth Quintile** | | | | | | | | |
| **Q1 - Poorest** (n=21) | 8 (38.1%) | 4.8 (1.6 – 14.1) | 6 (35.3%) | 2.77 (1.0 – 7.4) | 16 (76.2%) | 15.6 (3.9 – 61.8) | 9 (52.9%) | 2.6 (1.3 – 5.4) |
| **Q2** (n=38) | 12 (29.3%) | 3.9 (1.4 – 11.3) | 7 (20.6%) | 1.61 (0.6 – 4.4) | 20 (52.6%) | 10.8 (2.7 – 43.2) | 13 (38.2%) | 1.9 (0.9 – 3.9) |
| **Q3** (n=50) | 8 (16.0%) | 2.0 (0.6 – 6.2) | 6 (12.5%) | Ref | 19 (38.0%) | 7.8 (1.9 – 31.6) | 13 (27.7%) | 1.4 (0.7 – 2.8) |
| **Q4** (n=50) | 4 (8.0%) | Ref | 0 | - | 12 (24.0%) | 4.9 (1.2 – 20.8) | 10 (20.0%) | Ref |
| **Q5*- Wealthiest** (n=41) | 0 | - | 0 | - | 2 (4.9%) | Ref | 0 | - |
| **Geographical residence** | | | | | | | | |
| **Urban*** (n=29) | 2 (6.9%) | Ref | 1 (3.7%) | Ref | 2 (6.9%) | Ref | 1 (3.7%) | Ref |
| **Rural** (n=171) | 30 (17.5%) | 2.5 (0.6 – 10.1) | 18 (11.3%) | 3.0 (0.4 – 21.9) | 67 (39.2%) | 5.7 (1.5 – 22.0) | 44 (27.5%) | 7.4 (1.1 – 51.9) |
| **Maternal education** | | | | | | | | |
| **None / early**  **childhood** (n=53) | 13 (28.9%) | 3.0 (1.3 – 6.7) | 8 (21.1%) | 5.7 (1.6 – 20.6) | 28 (62.2%) | 3.7 (2.2 – 6.3) | 18 (47.4%) | 3.5 (1.8 – 6.7) |
| **Primary** (n=83) | 11 (17.7%) | 1.8 (0.8 – 4.3) | 8 (13.8%) | 3.8 (1.0 – 13.7) | 24 (38.7%) | 2.3 (1.3 – 4.1) | 14 (24.1%) | 1.8 (0.9 – 3.7) |
| **Secondary or higher** (n=248) | 8 (9.6%) | Ref | 3 (3.7%) | Ref | 14 (16.9%) | Ref | 11 (13.4%) | Ref |
| **Ethno-linguistic Group** | | | | | | | | |
| **Lao-Tai*** (n=168) | 19 (11.3%) | Ref | 13 (8.2%) | Ref | 48 (28.6%) | Ref | 30 (19.0%) | Ref |
| **Mon-Khmer** (n=29) | 11 (37.9%) | 3.3 (1.8 – 6.3) | 5 (19.2%) | 2.3 (0.9 – 6.0) | 19 (65.5%) | 2.3 (1.6 – 3.3) | 12 (46.2%) | 2.4 (1.4 – 4.1) |
| **Hmong-Mien** (n=0) | N/A | N/A | N/A | N/A | N/A | N/A | N/A | N/A |
| **Chinese-Tibetan** (n=-0) | N/A | N/A | N/A | N/A | N/A | N/A | N/A | N/A |
| **Other** (n=3) | 2 (66.7%) | 5.9 (2.4 – 14.6) | 1 (33.3%) | 4.0 (0.7 – 21.9) | 2 (66.7%) | 2.3 (1.0 – 5.4) | 3 (100%) | 5.3 (3.8 – 7.3) |

USD = United States Dollar; LAK = Lao Kip

*Reference group

^#^Due to non-convergence, RR estimated using Poisson regression with robust standard errors
