# Supplementary material for: Financial burden of severe childhood illness on households in Lao People’s Democratic Republic: A prospective cohort study
Source: PLOS Glob Public Health. 2026 Feb 20;6(2):e0004783. doi: 10.1371/journal.pgph.0004783 (PMC12923058; doi:10.1371/journal.pgph.0004783)
Supplement: S7 Table — *Visit 1 = at enrolment during hospital admission; Visit 2 = at hospital discharge; Visit 3 = 2 weeks post hospital discharge; Visit 4 = 2 months post hospital discharge. (DOCX) [file pgph.0004783.s009.docx]

**S7 Table: Reported financial coping strategies to pay for out-of-pocket costs associated with severe illness by hospital**

|  | **National Children’s Hospital** | | | | **Salavan Provincial Hospital** | | | |
| --- | --- | --- | --- | --- | --- | --- | --- | --- |
|  | Visit 1*  (n=200) | Visit 2*  (n=196) | Visit 3*  (n=194) | Visit 4*  (n=185) | Visit 1*  (n=200) | Visit 2*  (n=200) | Visit 3*  (n=199) | Visit 4*  (n=186) |
| **Health insurance** | 40 (20%) | 37 (18.9%) | 7 (3.6%) | 7 (3.8%) | 199 (99.5%) | 200 (100%) | 24 (12.1%) | 50 (26.9%) |
| **Used savings** | 200 (100%) | 196 (100%) | 76 (39.2%) | 102 (55.1%) | 198 (99%) | 198 (99%) | 41 (20.6%) | 78 (41.9%) |
| **Borrowed money from relatives/ friends** | 22 (11%) | 35 (17.9%) | 5 (2.6%) | 6 (3.2%) | 46  (23%) | 29 (14.5%) | 7 (3.5%) | 4 (2.2%) |
| **Borrowed money from bank** | 0% | 2 (1%) | 0% | 1 (0.5%) | 3  (1.5%) | 0% | 0% | 1 (0.5%) |
| **Sold assets** | 0% | 1 (0.5%) | 1 (0.5%) | 1 (0.5%) | 2  (1%) | 1 (0.5%) | 0% | 0% |
| **Reduced household expenses** | 5 (2.5%) | 6  (3.1%) | 5 (2.6%) | 1 (0.5%) | 0% | 0% | 0% | 0% |
| **Delayed plans** | 1 (0.5%) | 1  (0.5%) | 0% | 0% | 0% | 0% | 0% | 0% |
| **Other** | 0% | 0% | 0% | 0% | 0% | 0% | 0% | 0% |

*Visit 1 = at enrolment during hospital admission; Visit 2 = at hospital discharge; Visit 3 = 2 weeks post hospital discharge; Visit 4 = 2 months post hospital discharge
